# Functional characterization of fungal lytic polysaccharide monooxygenases for cellulose surface oxidation

**DOI:** 10.1186/s13068-023-02383-3

**Published:** 2023-09-07

**Authors:** Yann Mathieu, Olanrewaju Raji, Annie Bellemare, Marcos Di Falco, Thi Truc Minh Nguyen, Alexander Holm Viborg, Adrian Tsang, Emma Master, Harry Brumer

**Affiliations:** 1 Michael Smith Laboratories, University of British Columbia, 2185 East Mall, Vancouver, BC V6T 1Z4 Canada; 2 BioProducts Institute, University of British Columbia, 2385 East Mall, Vancouver, BC V6T 1Z4 Canada; 3 Department of Chemical Engineering and Applied Chemistry, University of Toronto, 200 College Street, Toronto, ON M5S 3E5 Canada; 4 Centre for Structural & Functional Genomics, Concordia University, 7141 Sherbrooke-West Street, Montreal, H4B 1R6 Canada; 5 Department of Bioproducts and Biosystems, Aalto University, Kemistintie 1, 02150 Espoo, Finland; 6 Department of Chemistry, University of British Columbia, 2036 Main Mall, Vancouver, BC V6T 1Z1 Canada; 7 Department of Biochemistry and Molecular Biology, University of British Columbia, 2350 Health Sciences Mall, Vancouver, BC V6T 1Z3 Canada; 8 Department of Botany, University of British Columbia, 3200 University Boulevard, Vancouver, BC V6T 1Z4 Canada

## Abstract

**Background:**

Microbial lytic polysaccharide monooxygenases (LPMOs) cleave diverse biomass polysaccharides, including cellulose and hemicelluloses, by initial oxidation at C1 or C4 of glycan chains. Within the Carbohydrate-Active Enzymes (CAZy) classification, Auxiliary Activity Family 9 (AA9) comprises the first and largest group of fungal LPMOs, which are often also found in tandem with non-catalytic carbohydrate-binding modules (CBMs). LPMOs originally attracted attention for their ability to potentiate complete biomass deconstruction to monosaccharides. More recently, LPMOs have been applied for selective surface modification of insoluble cellulose and chitin.

**Results:**

To further explore the catalytic diversity of AA9 LPMOs, over 17,000 sequences were extracted from public databases, filtered, and used to construct a sequence similarity network (SSN) comprising 33 phylogenetically supported clusters. From these, 32 targets were produced successfully in the industrial filamentous fungus *Aspergillus niger*, 25 of which produced detectable LPMO activity. Detailed biochemical characterization of the eight most highly produced targets revealed individual C1, C4, and mixed C1/C4 regiospecificities of cellulose surface oxidation, different redox co-substrate preferences, and CBM targeting effects. Specifically, the presence of a CBM correlated with increased formation of soluble oxidized products and a more localized pattern of surface oxidation, as indicated by carbonyl-specific fluorescent labeling. On the other hand, LPMOs without native CBMs were associated with minimal release of soluble products and comparatively dispersed oxidation pattern.

**Conclusions:**

This work provides insight into the structural and functional diversity of LPMOs, and highlights the need for further detailed characterization of individual enzymes to identify those best suited for cellulose saccharification versus surface functionalization toward biomaterials applications.

**Supplementary Information:**

The online version contains supplementary material available at 10.1186/s13068-023-02383-3.

## Background

Biomass is a promising alternative to petroleum-based feedstocks for the production of valuable fuels, chemicals, and materials. Terrestrial plant biomass, in particular, is an abundant and renewable source of both complex carbohydrates and hydrocarbons (i.e., polysaccharides, lignin, and low molecular weight extractives) [1–5]. The development of chemical and enzymatic processes to convert lignocellulose into value-added products continues to expand, especially over the past decade [6–9]. However, the fractionation and transformation of lignocellulose remains challenging, due to its inherently heterogenous and recalcitrant nature [10–12]. Significant inspiration for the development of efficient biocatalytic methods to overcome these challenges comes from the microbial world: Diverse bacteria and fungi have evolved specialized hydrolytic and oxidative enzymatic systems to target individual biomass polymers for nutrient acquisition [13–20].

Lytic polysaccharide monooxygenases (LPMOs) have attracted attention for biomass deconstruction and biofuels production, due to their ability to potentiate the activity of cellulases and thereby enhance lignocellulose saccharification [21–24]. More recently, LPMOs have been harnessed for nanocellulose production [25–31] and the surgical introduction of carboxylate groups as reactive handles on cellulose and chitin surfaces [32, 33]. LPMOs are mononuclear copper enzymes, which have been classified into eight sequence-related Auxiliary Activity families (AA9, AA10, AA11, AA13, AA14, AA15, AA16 and AA17) in the Carbohydrate-Active Enzymes (CAZy) database [34–36]. First identified due to their ability to oxidatively cleave insoluble cellulose or chitin, which remain the predominant reported activities, certain LPMOs preferentially target xyloglucan [37], pectin [36] and starch [38]. LPMOs use molecular oxygen or hydrogen peroxide [39, 40] and external electron donors originating from enzymatic or non-enzymatic reactions to perform C–H bond activation leading to hydroxylation of C1 or C4 of glycosyl residues within glycan chains. Subsequent elimination results in chain cleavage and production of the corresponding C1 lactone (which may hydrolyze to the carboxylic acid) or C4 ketone (which may hydrate to the diol), respectively [41]. Based on this regioselectivity, LPMOs can be divided into three types: C1-oxidizing, C4-oxidizing, and C1/C4-oxidizing [42, 43].

Structurally, LPMOs are small β-sheet proteins that tightly coordinate the catalytic copper in a highly conserved histidine-brace motif [44]. LPMOs can also comprise multi-modular architectures containing non-catalytic carbohydrate-binding modules (CBM, e.g., CBM1 or CBM18, specific for cellulose and chitin, respectively), or other modules of unknown function [45, 46]. CBMs can enhance catalysis by diverse carbohydrate-active enzymes (CAZymes) through substrate targeting, particularly at low substrate consistency [47–49]. Notably, the deletion or replacement of native CBMs with those from different CBM families was detrimental to LPMO activity on insoluble substrates [28, 50–54].

AA9 comprises the first, and currently largest, family of fungal LPMOs, which has a rich history of study [34, 55]. The large size of AA9 and apparent sequence diversity of its members pose a challenge for the comprehensive, definitive functional characterization of this family. Here, we sought to increase the biochemical coverage of AA9 to enable more confident bioinformatic prediction of function within the family. We mined over 17,000 AA9 homologs from publicly available databases and used Sequence Similarity Network analysis to guide target selection from distinct clusters. Ultimately, 32 AA9-encoding genes were successfully produced heterologously in *Aspergillus niger* and LPMO activity was confirmed by HPAEC-PAD for 25 targets. Eight of these were chosen for purification and detailed biochemical characterization, with emphasis on cellulose functionalization through the introduction of C1 carboxylate and/or C4-keto groups and direct visualization of activity on cellulose surfaces using fluorescent labeling.

## Results and discussion

### Selection and production of AA9 LPMO targets

**Fig. 1:**
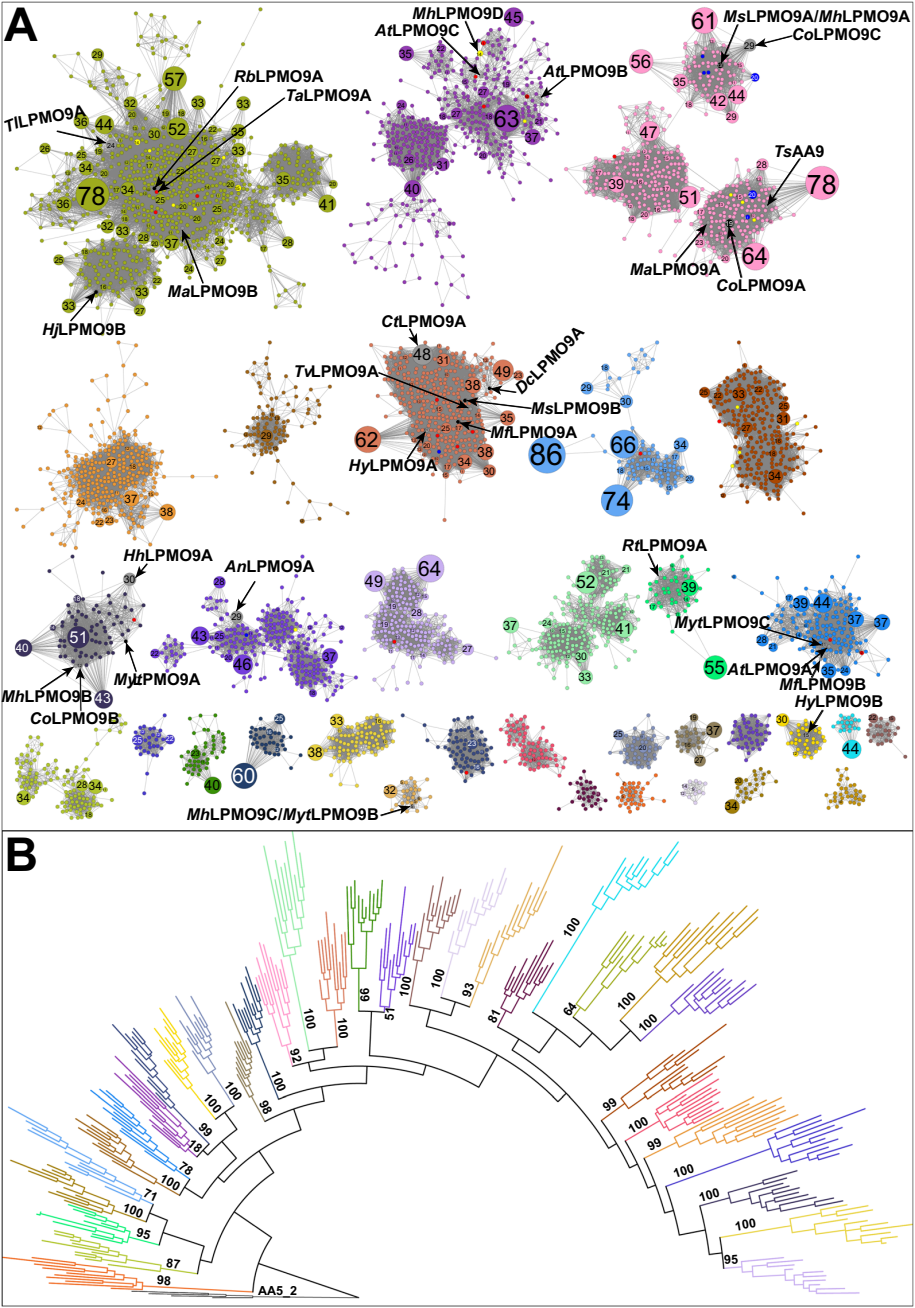
Sequence relationship of 5,328 AA9 catalytic modules. **A** Sequence similarity network (SSN) created with SSNpipe [58] and displayed in Cytoscape with yFiles Organic layout (Shannon et al. 2003a). Each node corresponds to one of the curated 5328 catalytic modules used as an input to build the SSN. Edges represent an alignment bit-score threshold of 250 that clusters the sequences into subgroups. AA9 members whose regioselectivity is available are colored in blue, yellow and red for C1, C4 and C1/C4 oxidizing enzymes, respectively (functional annotations were obtained from the CAZy database [20]). Sequences expressed in *Aspergillus niger* are colored in black. **B** Maximum-likelihood phylogenetic tree. 10 representative sequences for each subgroup defined by the sequence similarity network in **A**. Bootstrap values based on 100 replicates are shown

After removing identical sequences, and those with an arginine as the first amino acid instead of the catalytically essential histidine [56], the 17,575 AA9 homologs collected from several sequence databases were reduced to 5328 unique sequences. Scalable Sequence Similarity Network (SSN) analysis [57, 58] based on pairwise BLASTP analysis defined 33 subgroups (clusters) of AA9 modules using a bit-score threshold of 250 (Fig. 1). The choice of this threshold was based on inspection of the number of clusters and the number of sequences within them. A bit-score threshold of 225 produced fewer clusters (25 in total); however, some clusters were not well resolved. On the other hand, application of a bit-score threshold of 275 doubled the number of clusters from 33 to 66. To further validate this delineation, a maximum-likelihood phylogenetic tree was constructed from 10 sequences randomly selected from each of the 33 SSN clusters. The monophyletic groups inferred from the phylogenetic tree were identical to the subfamilies identified in the SSN and were color coded accordingly (Fig. 1). Importantly, we could correlate SSN clusters and phylogenetic groups using a bit-score threshold of 250, while we could not with thresholds of 225 or 275. Eleven of the 33 SSN clusters contained at least 1 characterized member.

From a broad selection of 404 targets spanning the major SSN clusters, the production campaign yielded 32 LPMOs from 10 clusters that were produced at quantities detectable by SDS-PAGE (Additional file 2: Table S1). These targets represent the ascomycete classes Sordariomycetes, Eurotiomycetes, and Dothideomycetes. Initial product analysis by HPAEC-PAD, using phosphoric acid-swollen cellulose (PASC) as a substrate and ascorbate as a reducing agent, indicated that 14 of these generated C1-oxidized products, 5 produced C4-oxidized products, 6 produced both C1 and C4-oxidized products, and 7 were apparently inactive (Additional file 2: Table S1), despite possessing intact histidine brace active-site motifs [56] (see the recent review by Vandhana et al. for an in-depth discussion of the potential functions of non-canonical LPMO-like proteins [59]). Of the 25 active LPMOs identified herein, 8 were selected for detailed characterization based on clear and detectable activity, and success in protein production and purification (Additional file 2: Table S1): *Co*LPMO9A, *Dc*LPMO9A, *Hj*LPMO9B, *Mf*LPMO9A, *Ms*LPMO9A, *Ms*LPMO9B, *Myt*LPMO9A, and *Rb*LPMO9A. To our knowledge, except for *Hj*LPMO9B, all selected sequences represented the first LPMO to be characterized from the source organism. Like *Hj*LPMO9A (formerly *Tr*Cel61A [53]), *Hj*LPMO9B displayed C1/C4-oxidizing activity; however, it does not possess a CBM. *Myt*LPMO9A shares 91% sequence similarity with the previously characterized *Mt*LPMO9D [60] and both display C1-oxidizing activity. *Myt*LPMO9A is from *Myriococcum thermophilum* and is referred to here with a three letter acronym denoting the source organism to distinguish it from *Mt*LPMOs that originate from *Myceliophthora thermophila*.

### Impact of cellulose type on LPMO activity

**Fig. 2:**
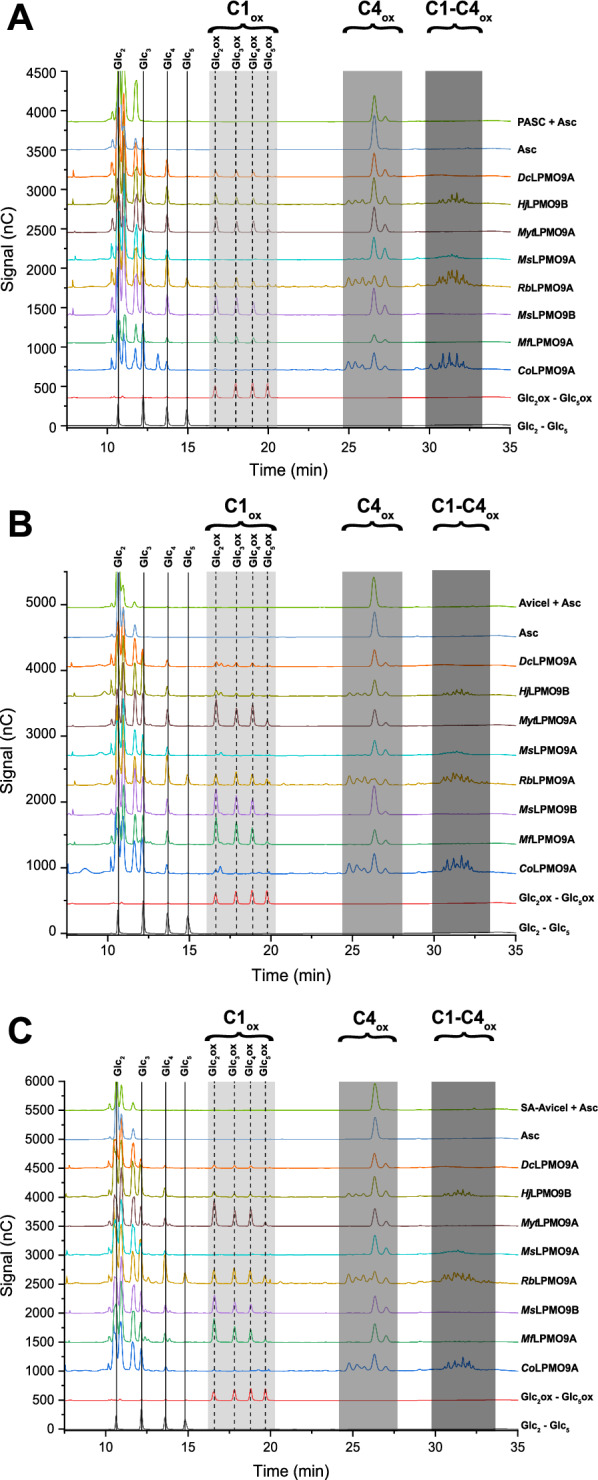
HPAEC-PAD analysis of LPMO regioselectivity towards three cellulosic substrates. Chromatograms over the retention times of 7.5–35 min. Native and C1-oxidized cello-oligosaccharide standard peaks are annotated. All activity assays were conducted using 5 µM of enzyme, 0.1% PASC (**A**), 1% Avicel (**B**) or 1% SA-Avicel (**C**) and 1 mM ascorbic acid for 16 h at 50 °C. The negative controls (substrate + electron donor) contained all assay components except enzyme

**Fig. 3:**
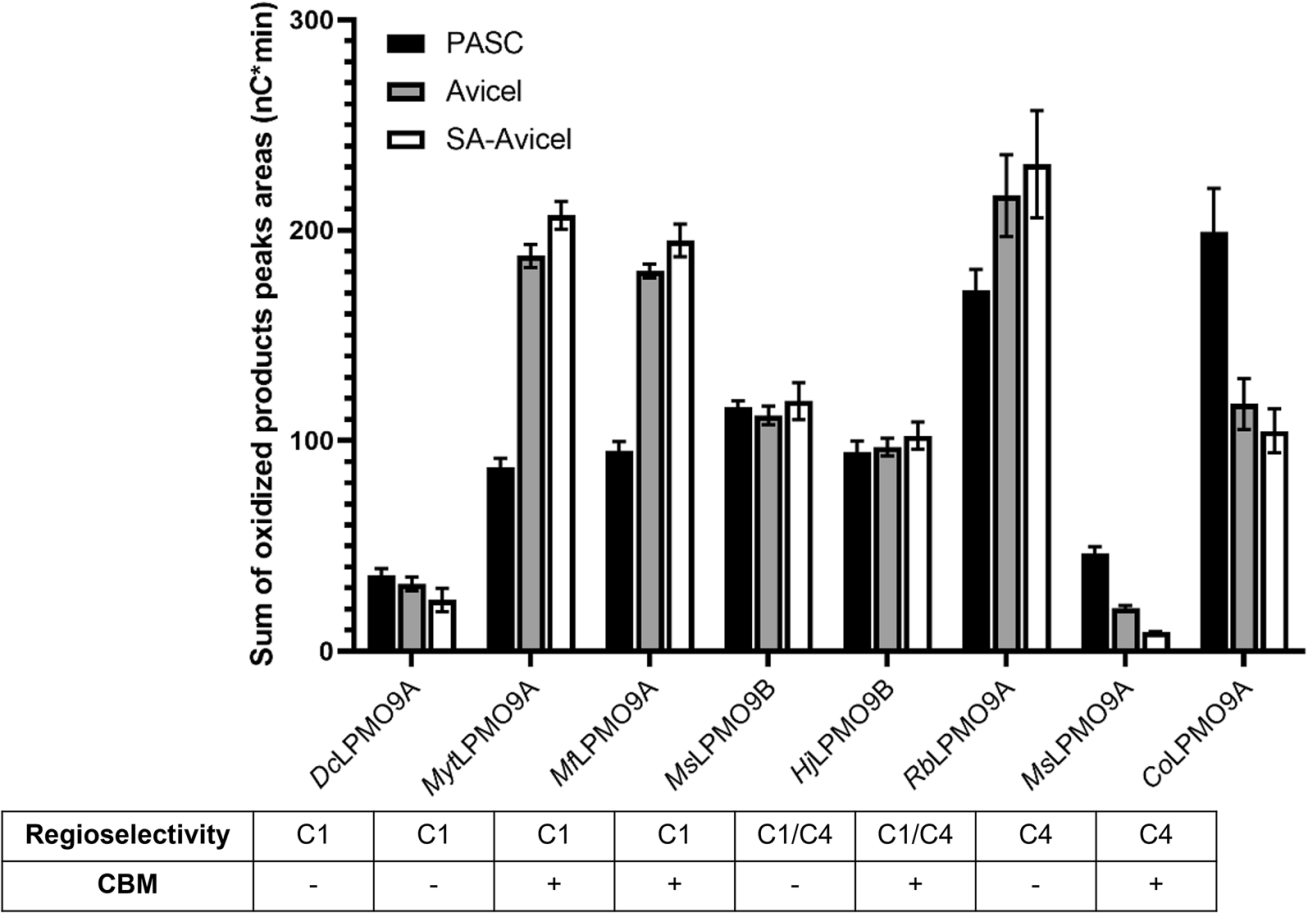
Activity of 5 µM LPMOs after 16 h on PASC (0.1%), Avicel (1%), and SA-Avicel (1%) with 1 mM ascorbic acid as an electron donor. Semi-quantitative analysis of C1 and C4 oxidized products was completed by summing the peak areas of C1-oxidized and C4-oxidized oligosaccharides. Each bar is the average of three independent assays measured singly by HPAEC-PAD, with error bars indicating the standard error of the mean

Following the activity screens using PASC (Additional file 2: Table S1), the eight purified LPMOs were compared using both PASC and Avicel as cellulose substrates having different crystallinity. Sulphanilic acid-treated Avicel (SA-Avicel) was also included in the comparative analysis as this derivatized form of Avicel would be later used for fluorescent labeling studies. *Mf*LPMO9A, *Ms*LPMO9B, *Myt*LPMO9A and *Dc*LPMO9A consistently released C1-oxidized cello-oligosaccharides from PASC, Avicel, and SA-Avicel (Fig. 2). By contrast, peaks corresponding to C4-oxidized products were consistently detected when treating the cellulosic substrates with *Co*LPMO9A and *Ms*LPMO9A, whereas *Rb*LPMO9A and *Hj*LPMO9B consistently produced both C1 and C4-oxidized products (Fig. 2). Commensurate with previous studies [61], non-oxidized cello-oligosaccharides were also released by all LPMOs. To simplify analyses, total peak areas of all C1-oxidized products, all C4-oxidized products, and all C1/C4-oxidized products were used as semi-quantitative estimates of cellulose deconstruction following overnight reactions (Fig. 3). Notably, both *Dc*LPMO9A and *Ms*LPMO9A lack a CBM and generated lowest amounts of soluble products from all substrates. Crucially, pre-treatment of Avicel with sulphanilic acid to block intrinsic carboxylate groups did not alter LPMO regioselectivities nor significantly alter LPMO performance.

Among the eight LPMOs biochemically characterized herein, the CBM-containing LPMOs generally released more soluble products regardless of substrate type (Fig. 3). The exception was *Myt*LPMOA, which lacks a CBM and yet performed similar to CBM-containing LPMOs tested in this study. Of note, low (0.1–1%) cellulose consistency was used in this study and similar impacts of cellulose binding modules on LPMOs with different regioselectivity were previously reported [62, 63].

### Impact of reducing agent on LPMO activity

**Fig. 4:**
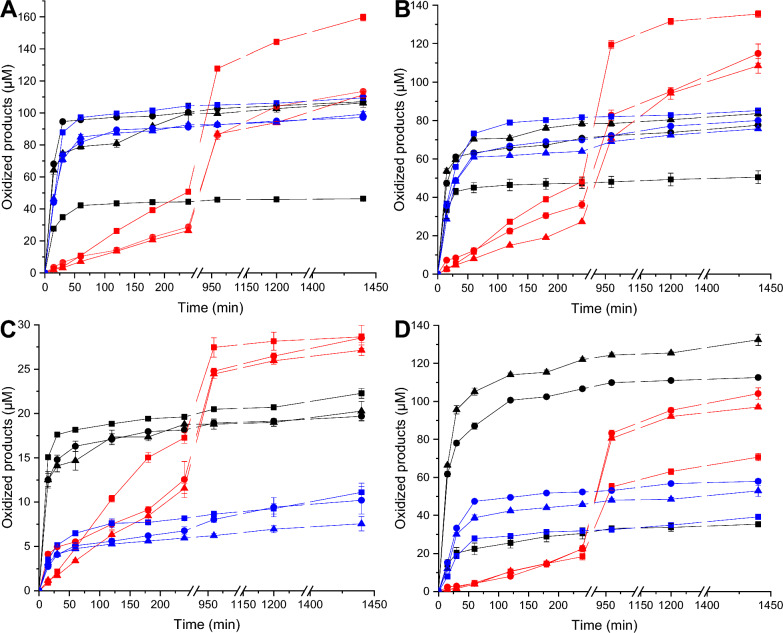
Time-course release of soluble oxidized products for **A** *Mf*LPMO9A, **B** *Ms*LPMO9B, **C** *Dc*LPMO9A, and **D** *Myt*LPMO9A. For each panel, 5 µM LPMOs were incubated at 50 °C for 24 h with 1 mM of either ascorbic acid (black lines), gallic acid (red lines) or cysteine (blue lines) with PASC (squares), Avicel (circles) or SA-Avicel (triangles). For each time point, *T. reesei* cellulase cocktail was used to convert all C1-oxidized products into cellobionic acid and quantified as the total C1-oxidized ends generated (µM). Total oxidized ends were obtained by quantifying cellobionic acid by HPAEC-PAD against a standard curve. Every point is the average of three independent assays measured individually by HPAEC-PAD, with error bars indicating the standard error of the mean

Ascorbic acid, gallic acid, and cysteine have previously been shown to exhibit differential reactivity as small-molecule electron donors for LPMOs [64, 65]. Hence, the impact of electron donor on the LPMOs of the present study was evaluated. Due to a lack of pure standards to enable quantitation of C4 products, only C1-oxidizing LPMOs were used for time-course investigations. For the four C1-oxidizing LPMOs, product release from all cellulose substrates approached maximum levels after 2 h when using either ascorbic acid or l-cysteine as the reducing agent (Fig. 4). By comparison, soluble product formation was slower in reactions amended with gallic acid, in which maximum product release was measured after 16 h (Fig. 4). Detailed consideration of the time-course curves for oxidized soluble product formation by each C1 LPMO revealed nuanced impacts of electron donor and cellulose substrate combinations. For example, *Mf*LPMO9A released highest levels of soluble product from PASC when using gallic acid as the reductant, whereas using gallic acid reduced total soluble product released from Avicel by 30%. Instead, both *Mf*LPMO9A and *Ms*LPMO9B released highest levels of soluble product from Avicel when using ascorbic acid as the reductant, whereas using ascorbic acid reduced total soluble products from PASC by over 55%. Notably, the choice of electron donor did not substantially impact soluble product release by *Dc*LPMO9A from any of the cellulose substrates, and using cysteine as reductant did not substantially change the LPMO activities on the different cellulose types.

Previous studies investigating the impact of reductant on soluble product release from amorphous cellulose compared three family AA9 LPMOs from *Myceliophthora thermophila* that display different regioselectivities, and in all cases, the preferred reductant was ascorbic acid, followed by gallic acid and then L-cysteine [64]. Conversely, among closely related chitin-active AA10s from *Cellulomonas* species, cysteine, a sulfur containing electron donor, generated more oxidized soluble products [65]. In our case, the time-course experiments clearly demonstrated that each enzyme favored a specific electron donor/substrate couple for the release of soluble C1-oxidized products. In addition, the protein modularity and structure also impact substrate-dependent preferences for particular reducing agents. Altogether, a growing body of literature, including our present analysis, demonstrates that each LPMO needs to be carefully assessed in light of specific applications (total versus controlled oxidation), with regard to activity and reducing agent specificity for a particular substrate. For instance, using two different electron donors on the same substrate can result in different rates of oxidation and hence surface modification vs. erosion.

### LPMO action on cellulose fiber surfaces

**Fig. 5:**
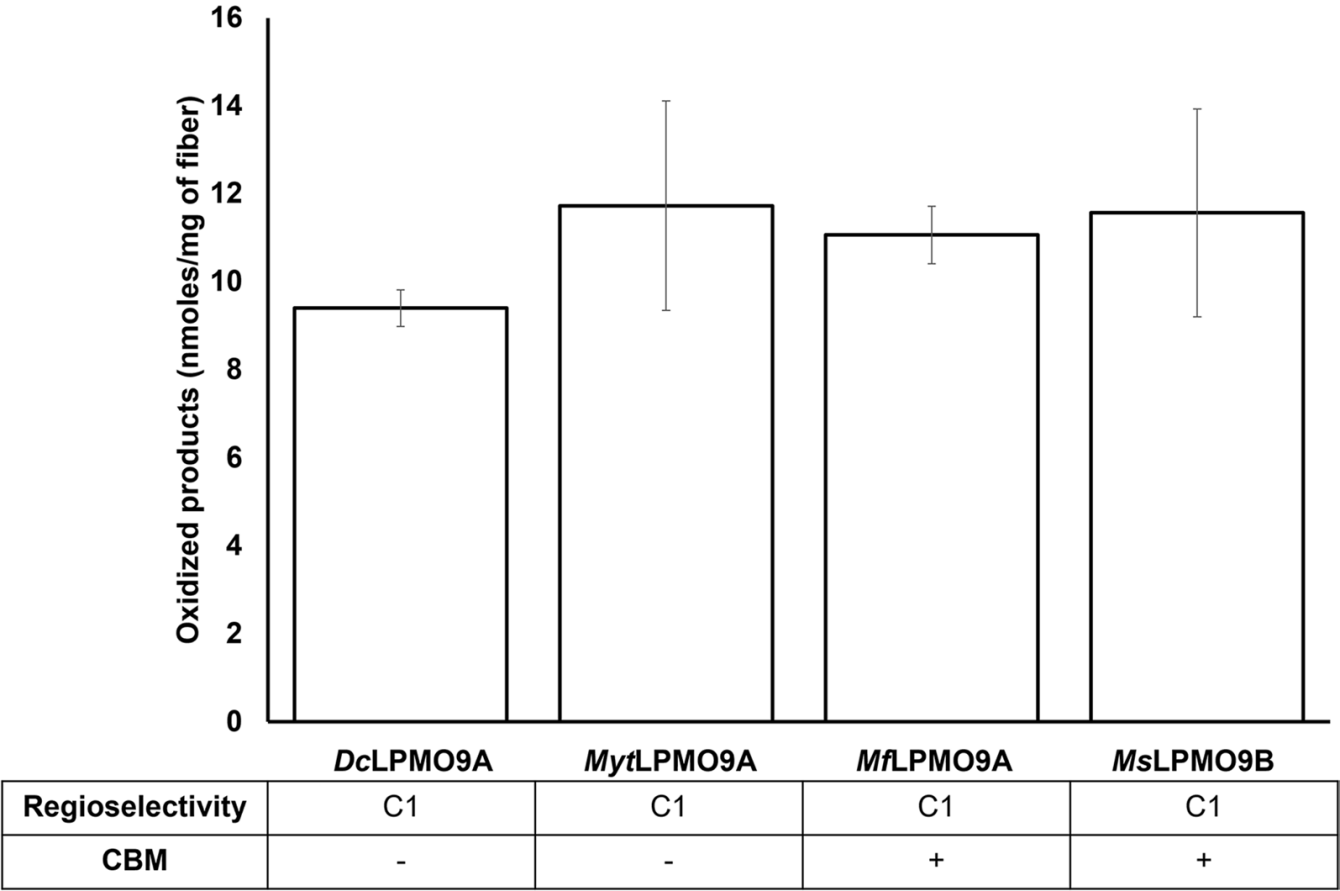
Assessing LPMO (5 µM) activity on SA-Avicel (1%) fiber after 16 h with 1 mM ascorbic acid as an electron donor. *T. reesei* cellulase cocktail was used to convert all oxidized insoluble products into cellobionic acid and quantify the total C1-oxidized ends introduced to the fiber (nanomoles per mg of starting fiber). Total oxidized ends were obtained by quantifying cellobionic acid by HPAEC-PAD against a standard curve. Each bar is the average of three independent assays measured singly by HPAEC-PAD with error bars indicating the standard error of the mean

SA-Avicel was prepared to block pre-existing carboxyl groups in the cellulose substrate [66] and then used to investigate LPMO-mediated oxidation of cellulose fiber surfaces. Whereas the quantitation of carbonyls introduced by C4 LPMOs remains challenging, carbonyls introduced by C1 LPMO oxidation were quantified through enzymatic hydrolysis of the residual cellulose to glucose, cellobiose and cellobionic acid. Regardless of whether the C1 LPMO comprised a CBM, between 9 and 12 nmol of carboxylate groups per mg of starting material were introduced to the fiber surface (Fig. 5). For material applications requiring fiber surface oxidation, preferred LPMOs would be those that achieve high surface oxidation while minimizing loss in cellulose yield (mass) through release of soluble products [32, 33]. Such a product profile was achieved using *Dc*LPMO9A where carboxylate groups on the fiber surface were comparable to the other LPMOs while retaining 2–4 times higher fiber yield (Additional file 1: Figure S1).

**Fig. 6:**
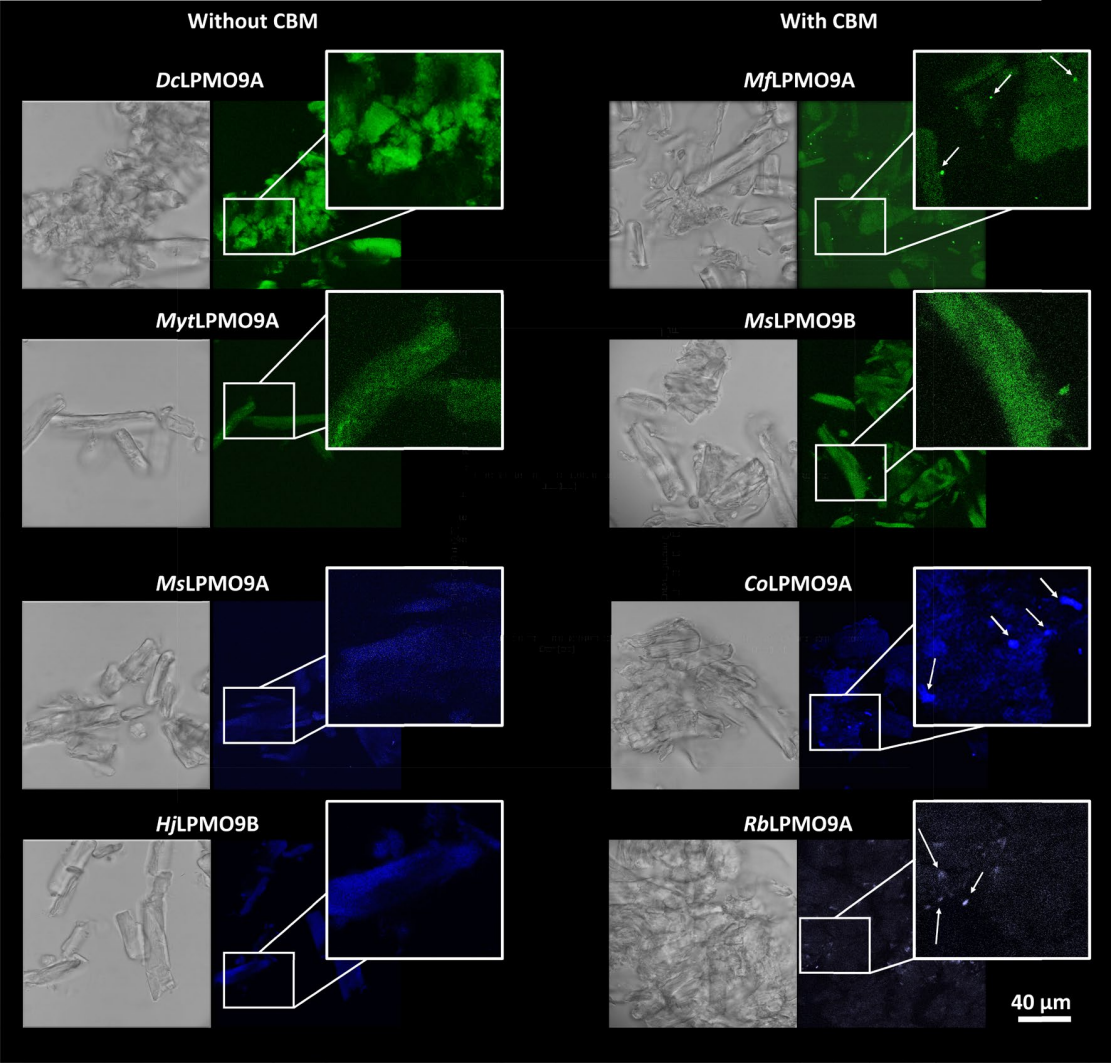
Brightfield and confocal images of LPMO-treated SA-Avicel labeled using cyanine aminooxy dye (blue) for C4 products and rhodamine chloride (green) for C1 products. For each panel, 5 µM LPMOs were incubated at 50 °C for 24 h with 1% SA-Avicel and 1 mM of gallic acid. Insoluble products were separated, labeled with fluorescent dye, and visualized using a confocal microscope. Untreated substrates did not show fluorescence signals (Additional file 1: Figure S2)

To provide additional insight into the distribution of LPMO activity on fiber surfaces, the rhodamine green-fluorescent dye and cyanine 647 aminooxy dye were used to label the enzymatically introduced C1 carboxylate and C4-keto groups, respectively. Although attempts to quantify the resulting fluorescence were unreliable due to the unavoidable heterogeneity of the substrate suspensions, the pattern of LPMO activity on SA-Avicel surfaces could be visualized by confocal microscopy. Oxidations introduced by all LPMOs lacking a CBM (i.e., *Dc*LPMO9A, *Myt*LPMO9A, *Ms*LPMO9A, and *Hj*LPMO9B) were evenly dispersed across the fiber surface (Fig. 6); by comparison, the presence of a CBM in *Mf*LPMO9A, *Co*LPMO9A, and *Rb*LPMO9B led to a more punctate distribution of oxidized sites across the cellulose fiber surface (Fig. 6). *Ms*LPMO9B also comprises a CBM1 domain, however, the oxidation pattern on fiber surfaces was similar to that introduced by the LPMOs lacking a CBM (Fig. 6).

The overall trend whereby CBM-containing LPMOs introduce punctate oxidation sites on cellulose fiber surfaces agrees with earlier predictions drawn from biochemical and dynamic modeling studies of *Sc*LPMO10C and a truncated variant lacking the CBM [54]. These authors suggested that the presence of the CBM promotes multiple and localized ScLPMO10C action on the cellulose substrate. Likewise, our data provide visual confirmation of the significant impacts that CBMs have on LPMO action.

To investigate the relative distribution of C1 and C4 oxidized sites introduced by C1/C4 type LPMOs, rhodamine green and cyanine 647 aminooxy dyes were used together to label cellulose fibers after treatment with *Hj*LPMO9B and *Rb*LPMO9A. The order of the labeling did not influence the analysis; C1 labeling and C4 labeling can be performed simultaneously without cross-reactivity or inhibition of either labeling process. After labeling, cellulose treated with *Hj*LPMO9B or *Rb*LPMO9A revealed a blended distribution of the green and blue fluorescence, suggesting co-location of C1- and C4-oxidation events on the fiber surface regardless of the presence of a CBM. While this points to minimal impact of substrate surface characteristics on C1- versus C4-oxidation events, methods to quantify C4 oxidation on cellulose fiber surfaces are needed to confirm this interpretation.

## Conclusions

The present study investigated the impact of LPMO sequence, cellulose morphology, and small-molecule reductant on the soluble product profile, extent of substrate degradation, and pattern of cellulose surface oxidation. Although regiospecificity could not be correlated to SSN clusters, the extent of soluble product release and punctate pattern of cellulose surface oxidation was notably affected by the presence of a carbohydrate-binding module (CBM) in tandem. Of further practical relevance, the optimal reductant was LPMO-specific and influenced by cellulose type. The development of LPMOs as tools not only for cellulose deconstruction, but also to produce functionalized cellulosic materials relies on having a holistic view of LPMO action that tracks both soluble and insoluble product formation. The fluorescent labeling method applied herein not only allows us to qualitatively confirm surface oxidation of fibers, but also to visually observe the pattern of oxidation relevant for material applications. The analyses revealed that while *Myt*LPMO9A and CBM-containing LPMOs characterized herein might be the preferred enzymes for cellulose deconstruction, *Dc*LPMO9A and *Ms*LPMO9A would be preferred for dispersed cellulose surface oxidation leading to carboxyl and keto-functional groups, respectively. Applications benefiting from these surface modifications include fiber dye retention and fiber carding; the known ability to produce the relevant enzymes in an industrial production system (i.e., *Aspergillus niger*) motivates near term development of these application concepts.

## Materials and methods

### Materials

Avicel® was purchased from Sigma Aldrich (St. Louis, MO, USA), glucose was purchased from Fisher Scientific (Hampton, NH, USA), and cellobiose was purchased from Acros Organics (Morris Plains, NJ, USA). Cello-oligosaccharides (Glc2–Glc5) were purchased from Megazyme (Bray, Ireland). C1-oxidized cello-oligosaccharides standards were prepared using *Podospora anserina* cellobiose dehydrogenase (GenBank accession number: CAP64840) [67], kindly provided by Dr. Jean-Guy Berrin (INRA, Marseille, France). Briefly, 1 µM of CDH was added to 2 mM of each cello-oligosaccharide (Glc_2_-Glc_5_) in 50 mM sodium acetate buffer pH 5.0, followed by incubation at 37 °C. Full conversion was achieved by supplementation of additional CDH every 24 h for 3 days. Phosphoric acid-swollen cellulose (PASC) was prepared from Avicel® following a previously published protocol [68].

Treatment of Avicel with sulphanilic acid (SA-Avicel) was performed to block pre-existing carboxylic acid groups on Avicel. Briefly, Avicel was suspended in dimethylformamide (DMF) (Bioshop Ontario, Canada) at 50 g/L before adding the coupling reagent 12.5 mM benzotriazol-1-yloxytripyrrolidinophosphonium hexafluorophosphate (PyBOP) (Sigma Aldrich Co. Missouri, USA) and 200 mM N, N-di-isopropylethylamine (DIPEA) (Sigma Aldrich Co. Missouri, USA). After gentle mixing for 30 min, 100 mM of sulphanilic acid was added and the mixing continued for an additional 60 min. The reaction was centrifuged and washed several times with DMF to remove unbound sulphanilic acid. The SA-Avicel product was suspended in MilliQ water to a final concentration of 20 g/L.

### Bioinformatics for target selection

A total of 17,575 AA9 sequences were retrieved from Carbohydrate-Active enZYmes (CAZy; www.cazy.org), Joint Genome Institute (JGI; www.jgi.doe.gov), and Concordia Characterized Lignocellulose-Active Enzymes (CLAE; www.clae.fungalgenomics.ca) and Centre for Structural and Functional Genomics (CSFG; www.concordia.ca/research/genomics.html) databases. Where present, signal peptides and additional modules, such as carbohydrate-binding modules, were removed to isolate the catalytic modules for subsequent analyses. Catalytic modules sharing 100% identity were down-sampled to one sequence to eliminate redundancy. Moreover, obviously erroneous sequences generated by incorrect splicing predictions were removed via the identification of unusually long deletions/insertions through visual inspection of a multiple sequence alignment. Initial clustering of highly similar sequences was performed using CD-HIT [69] with an 80% identity cut-off [70], yielding a reduced dataset of 5,328 sequences from the original 17,575, and merged into meta-nodes. Sequence similarity networks (SSNs) were generated by computing all-versus-all pairwise local alignments of the 5,328 curated AA9 catalytic domains using SSNpipe [58], which generated the *E*-value, bit score, alignment length, sequence identity and sequence similarity for all sequence pairs. The data were filtered using a bit-score threshold of 250 to generate the final SSNs. To constitute a subfamily or cluster, connected clusters were required to contain at least 20 sequences from different organisms to avoid over-representation of closely related organisms. Members of each putative subfamily were identified using NetworkX [71] and SSNs were visualized with Cytoscape [72] using the yFiles organic layout.

For each cluster, 10 random sequences were aligned with MAFFT v7.402 using the L-INS-I algorithm [73] on the CIPRES Science Gateway (www.phylo.org) [74]. Four AA5_2 sequences (Genbank accessions AHA90705, EFQ30446, CAP96757 and XP_003719369) were included as an outgroup. The quality of the alignment was validated by visual inspection in AliView [75]. A maximum likelihood phylogenetic tree was estimated using RAxML v.8 [76] with 100 bootstrap replications on the CIPRES Science Gateway portal and formatted using iTOL [77].

### Gene cloning, transformation into *Aspergillus niger*

The AA9 sequences selected for expression in *Aspergillus niger* were PCR-amplified from cDNA or genomic DNA of source organisms (Additional file 2: Table S1). Where source organisms were not readily available, genes were synthesized (Integrated DNA Technologies Inc.; Iowa, USA). When obtained from the source organisms, the fungal strains were cultivated in 25 mL of Trametes defined medium (TDM) [78] supplemented with 2% of a mix of alfalfa and barley straws. *Hypocrea jecorina* cultures were incubated at 30 °C and all other fungi were incubated at 45 °C. In all cases, cultivations continued for 24 h with shaking at 220 rpm. Mycelia were harvested, ground into powder and genomic DNA was extracted as described previously [79]. In parallel, total RNA was extracted using the RNeasy® Plant Maxi Kit (Qiagen, USA) and complementary DNA (cDNA) was synthesized using the SuperscriptTM III reverse transcriptase (Invitrogen, ThermoFisher Scientific, USA) as per manufacturer instructions. The amplified or synthesized genes were cloned between the *A. niger* glucoamylase gene promoter and terminator of the plasmid ANIp4 [80] or its derivatives.

The recombinant LPMO genes were introduced into the *A. niger* genome by replacement of the glucoamylase gene (UniProt: A2QHE1) using CRISPR/Cas9 genome editing. The CRISPR plasmid ANEp8-Cas9-gRNA_glaA used in this study contains the guide RNA sequence 5’-GCAGTGTGACTGTCACCTCG-3’ that targets the coding region of the glucoamylase gene. The guide RNA was cloned into the plasmid ANEp8-Cas9-gRNA as described [81] to generate ANEp8-Cas9-gRNA_glaA. The CRISPR plasmid ANEp8-Cas9-gRNA_glaA along with a recombinant LPMO gene were introduced into *A. niger* by protoplast transformation [82, 83]. Strain CSFG_9047 (A1513 Δ*kusA* Δ[*prtT amyC agdA*] Δ*bglA* Δ*laeA*), engineered for this study, was derived from *A. niger* A1513 (Fungal Genetics Stock Center, USA), an industrial cell factory which is also designated as CBS 513.88 [84]. The gene models of the *A. niger* CBS 513.88 genome sequence [84] were used to guide the construction of the production host CSFG_9047; and the gene IDs of CBS 513.88 were used as references for the deleted genes (see below). Strain CSFG_9047 is a uridine/uracil prototroph (Δ*pyrG*; ID: An12g03570}; defective in non-homologous end-joining DNA repair (Δ*kusA*; ID: An15g02700) to promote homologous recombination [85]; deficient in extracellular proteases (Δ*prtT*; ID: An04g06940) to reduce proteolytic degradation of secreted proteins as the gene *prtT* encodes a regulator of extracellular proteases [86]; and defective in citric acid production (Δ*laeA*; ID: An01g12690) to prevent acidification of the culture medium [87]. During the course of this study, proteomic analysis detected a number of extracellular proteins in the culture filtrates. We removed the genes encoding three of them to reduce the level of background proteins: alpha-glucosidase (Δ*agdA*; ID: An04g06920); alpha amylase Δ*amyC*; ID: An04g06930); and beta-glucosidase (Δ*bglA*; ID: An18g03570). The deletion of the *bglA* gene is important for this study because the beta-glucosidase activity in the culture filtrate interferes with the LPMO activity assays.

### Protein production and purification

Selected *A. niger* transformants were inoculated in liquid modified minimal medium (MMJ) [88] supplemented with 0.1% arginine and screened for protein production by SDS-PAGE. Transformants producing the target proteins were cultured in 200 mL to 1 L liquid MMJ. Culture supernatants were concentrated and buffer-exchanged using Vivaflow® cassettes (Sartorius, Göttingen, Germany) based on the manufacturer’s protocol and according to [89]. Once exchanged to 50 mM sodium acetate (pH 5.0), recombinantly produced LPMOs were analyzed on Criterion™ TGX Stain-Free protein gels (Bio-Rad, Canada) and quantified by densitometry using GeneTools v.4.3.9.0 (Syngene, UK) according to the instructions provided by the manufacturer. Bovine serum albumin (BSA) was used as the standard for quantification and the identity of each LPMO was confirmed by mass spectrometry as described below.

The heterologously produced proteins were then tested for LPMO activity as described below. Supernatants showing activity were concentrated to 2 mL and filtered through a 0.22-µm PES syringe filter before purification. Protein purification was achieved using size exclusion chromatography on a 16-mm × 600 mm Superdex® 75 column pre-equilibrated with 50 mM sodium acetate pH 5.0 at 1 mL.min^−1^; eluted samples were collected in 500-µL fractions. The fractions were analyzed by SDS-PAGE and those containing the target protein with > 95% purity were pooled and concentrated before being flash frozen and stored at –80 °C. Protein concentrations were determined by measuring *A*_*280*_ using extinction coefficients which were calculated using the ProtParam tool on the Expasy server (http://web.expasy.org/protparam/).

### LC–MS/MS-based LPMO identification in culture supernatants

Two volumes of cold methanol were added to 100 µL of cleared culture supernatant and kept on ice for 30 min. Samples were centrifuged for 30 min at 15,000 × g and 4 °C. The precipitated protein pellet was washed once with 300 µL of cold 60% methanol in water before being suspended in 30 µL of 6 M urea solution containing 100 mM ammonium bicarbonate (pH 8.0) and digested with trypsin as previously described [90]. Aliquots of peptide digest were analyzed by liquid chromatography–tandem mass spectrometry (LC–MS/MS) using an Agilent 1260 Infinity II chromatography system connected in-line with a Thermo-Finnigan 7 Tesla LTQ-FT MS system. MS/MS data were processed for protein identification using the precursor ion quantitation workflow from Proteome Discoverer 2.4. MS/MS fragmentation data were queried against a database of 17,897 protein sequences comprising the *A. niger* NRRL3 protein models plus a collection of recombinant protein sequences, including the LPMOs of interest.

### Enzyme activity assays

Unless stated otherwise, all assays were performed in 1.7 mL tubes incubated in an Eppendorf Thermomixer C (Hamburg, Germany) at 50 °C with shaking at 1,000 rpm. For initial screens of culture supernatant, reactions (300 µL) were performed in 50 mM sodium acetate buffer (pH 5.0) containing 5 µM of enzyme, 0.1% w/v PASC, and 1 mM ascorbic acid; the ascorbic acid was added last to initiate the reaction. Purified enzymes were tested the same way using three substrates (0.1% w/v PASC, 1.0% w/v Avicel, and 1.0% w/v SA-Avicel) and three electron donors (ascorbic acid, gallic acid or l-cysteine). Reaction aliquots were collected at 15 min, 30 min, 60 min, 120 min, 240 min, 960 min, 1,200 min and 1,440 min. In all cases, reactions were filtered through a 0.2-µm cellulose acetate spin filter (VWR, P/N: 2994–752) for soluble products analysis.

Reaction products were analyzed by High Performance Anion Exchange Chromatography (HPAEC) using an ICS-5000 system (Dionex, Sunnyvale, CA, USA) coupled to a gold electrochemical detector (Dionex, P/N: 072044) for pulsed amperometric detection (PAD) [91, 92]. For initial screens of LPMO activity in culture supernatants, 25 µL of the reaction mixture were directly injected on a CarboPac PA1 (2 × 250 mm) analytical column (Dionex, P/N: 057178) and corresponding guard column (2 × 50 mm) (Dionex, P/N: 057179) maintained at 30 °C. Product separation was achieved at a constant flowrate of 0.25 mL.min^−1^ with the following method: initial column equilibration for 10 min with an isocratic flow of 0.1 M NaOH, followed by a linear gradient to 0.25 M NaOAc over 30 min and then a stepwise increase to 0.9 M NaOAc in 1 min. To quantify reaction products from purified LPMOs, reaction mixtures were filtered through a 0.2 µm cellulose acetate spin filters before adding 0.1 unit of a *T. reesei* cellulase cocktail (Celluclast, Sigma Aldrich, P/N: C2730-30) to 100 µL of the reaction filtrate and incubation overnight at 37 °C. In parallel, 100 µL of 50 mM sodium acetate buffer (pH 5.0) was added to the cellulose retentate before adding 0.1 unit of Celluclast and incubating the sample at 37 °C for 48 h. Following enzymatic digestion using Celluclast, 25 µL of sample were analyzed using HPAEC-PAD as described above. The resulting glucose, cellobiose and cellobionic acid products were quantified based on corresponding standards. In all cases, reactions were performed as triplicate independent experiments.

### Fluorescent labeling and confocal microscopy

To label the C1-oxidized positions in LPMO-treated SA-Avicel, the insoluble cellulose product was recovered by centrifugation at 14,000×*g* for 10 min and then coupled to Rhodamine 110 chloride (Rh110, Sigma Aldrich, St. Louis, MO, USA, P/N: 83,695; excitation max = 498 nm; emission max = 520 nm) using benzotriazol-1-yloxytripyrrolidinophosphonium hexafluorophosphate (PyBOP) and *N*,*N*-di-isopropylethylamine (DIPEA). Briefly, 4 mM of DIPEA and 1 mM of PyBOP in dimethylformamide (DMF) were gently mixed with the insoluble cellulose fraction (≤ 10 mg) for 5 min before adding 0.25 mM of Rh110, followed by gentle mixing for 5 min. The labeling process involves conventional amide bond formation, where the enzymatically introduced carboxylic acid group reacts with the amine group of Rh110. The reaction was protected from light to prevent bleaching of the fluorophore.

To label C4-oxidized positions in LPMO-treated celluloses, the residual insoluble cellulose fraction (≤ 10 mg) was treated with 0.25 mM cyanine 647 aminooxy dye (Biotium, Fremont, CA, USA; excitation max = 650 nm; emission max = 665 nm) in DMF for 5 min. The reaction was protected from light to prevent light bleaching of the fluorophore.

The reactions were centrifuged and washed multiple times with DMF to remove unbound dye from the labeled-insoluble product. Washes were monitored using an Infinite M200 spectrophotometer (Tecan, CHE) set to excitation and emission wavelengths of the corresponding fluorophores. Washes continued until unbound dye was not detectable in the washes. Labeled, insoluble products were suspended in 300 μL MilliQ water and stored away from light at room temperature.

A confocal microscope (Leica TCS SP5 Wetzlar, Germany) equipped with an argon laser at 20% power was used to image the labeled-insoluble products. Images were obtained using a 100 × oil-immersion lens with a FITC filter for Rh110 excitation at 480–495 nm and emission at 510–535 nm, while a Cy5 Filter for Cyanine 647 Aminooxy excitation at 635–655 nm and emission at 660–675 nm. At least four randomly chosen positions were analyzed per sample. Application Suite (LAS) was used for processing and annotation of captured images.

### Supplementary Information

**Additional file 1**: **Figure S1**: Product released by 5 μM C1 LPMOs after 16 h on PASC (0.1%), Avicel (1%), and SA-Avicel (1%)with 1 mM ascorbic acid as the electron donor. For each substrate, *T. reesei* cellulase cocktail was used to convert all C1-oxidized products into cellobionic acid, which was then quantified by HPAEC-PAD and reported as the total C1-oxidized ends generated (nanomoles per mg of starting fiber). Each bar is the average of three independent assays measured singly by HPAEC-PAD, with error bars indicating the standard error of the mean. **Figure S2**: Brightfield (A) and confocal (B) images of untreated SA-Avicel labelled using rhodamine chloride. 1% SA-Avicel with 1 mM of gallic acid was incubated at 50 °C for 24 h. Insoluble products were separated, labelled with fluorescent dye, and visualized using confocal microscopy. **Figure S3**: Process schematic for LPMO treatment of cellulosic substrates and subsequent soluble and insoluble product analysis. PASC (0.1%), Avicel (1%), and SA-Avicel (1%) were treated with LPMOs using either ascorbic acid, gallic acid, or cysteine as the electron donor. Insoluble products were separated from soluble products using centrifugation. For the soluble products analysis, HPAEC-PAD was used to annotate native and oxidized cello-oligosaccharide peaks. To quantify the C1-oxidized products, *T. reesei* cellulase cocktail was used to convert all C1-oxidized products into cellobionic acid, which was then quantified by HPAEC-PAD and reported as the total C1-oxidized ends generated (nanomoles per mg of starting fiber). For insoluble product analysis, separated insoluble products were labelled with either C1-specific or C4-specific fluorescent dye and subsequently visualized using confocal microscopy.

**Additional file 2**: Additional tables.

## Data Availability

All data and sequences supporting the conclusions of this article are included within the article and its additional files, deposited in public databases, or available from the authors upon reasonable request.

## Abbreviations

AA9: Auxiliary Activity Family 9
CAZyme: Carbohydrate-Active Enzyme
CBM: Carbohydrate-binding module
LPMO: Lytic polysaccharide monooxygenase
SSN: Sequence Similarity Network
