# Supplementary material for: Functional characterization of fungal lytic polysaccharide monooxygenases for cellulose surface oxidation
Source: Biotechnol Biofuels Bioprod. 2023 Sep 7;16:132. doi: 10.1186/s13068-023-02383-3 (PMC10486138; doi:10.1186/s13068-023-02383-3)
Supplement: Supplementary file 1 — Additional file 1: Figure S1: Product released by 5 μM C1 LPMOs after 16 h on PASC (0.1%), Avicel (1%), and SA-Avicel (1%)with 1 mM ascorbic acid as the electron donor. For each substrate, T. reesei cellulase cocktail was used to convert all C1-oxidized products into cellobionic acid, which was then quantified by HPAEC-PAD and reported as the total C1-oxidized ends generated (nanomoles per mg of starting fiber). Each bar is the average of three independent assays measured singly by HPAEC-PAD, with error bars indicating the standard error of the mean. Figure S2: Brightfield (A) and confocal (B) images of untreated SA-Avicel labelled using rhodamine chloride. 1% SA-Avicel with 1 mM of gallic acid was incubated at 50 °C for 24 h. Insoluble products were separated, labelled with fluorescent dye, and visualized using confocal microscopy. Figure S3: Process schematic for LPMO treatment of cellulosic substrates and subsequent soluble and insoluble product analysis. PASC (0.1%), Avicel (1%), and SA-Avicel (1%) were treated with LPMOs using either ascorbic acid, gallic acid, or cysteine as the electron donor. Insoluble products were separated from soluble products using centrifugation. For the soluble products analysis, HPAEC-PAD was used to annotate native and oxidized cello-oligosaccharide peaks. To quantify the C1-oxidized products, T. reesei cellulase cocktail was used to convert all C1-oxidized products into cellobionic acid, which was then quantified by HPAEC-PAD and reported as the total C1-oxidized ends generated (nanomoles per mg of starting fiber). For insoluble product analysis, separated insoluble products were labelled with either C1-specific or C4-specific fluorescent dye and subsequently visualized using confocal microscopy. [file 13068_2023_2383_MOESM1_ESM.pdf]

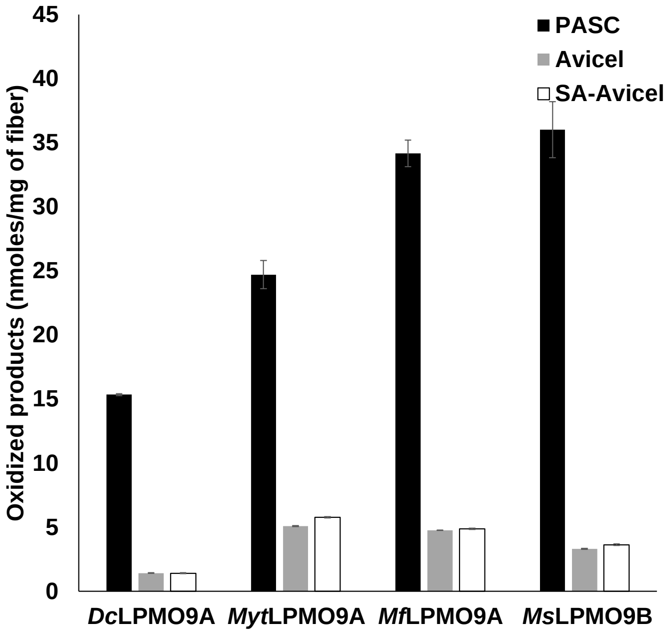

**Regioselectivity**	C1	C1	C1	C1
**CBM**	-	-	+	+

Figure S1: Activity of 5µM C1 LPMOs after 16 h on PASC (0.1 %), Avicel (1 %) and SA-Avicel (1 %) with 1 mM ascorbic acid as an electron donor. For each substrate, *T. reesei* cellulase cocktail was used to convert all C1-oxidized products into cellobionic acid and quantified as the total C1-oxidized ends generated (nanomoles per mg of starting fiber). Total oxidized ends were obtained by quantifying cellobionic acid by HPAEC-PAD against a standard curve. Every bar is the average of three independent assays measured singly by HPAEC-PAD, with error bars indicating the standard error of the mean.

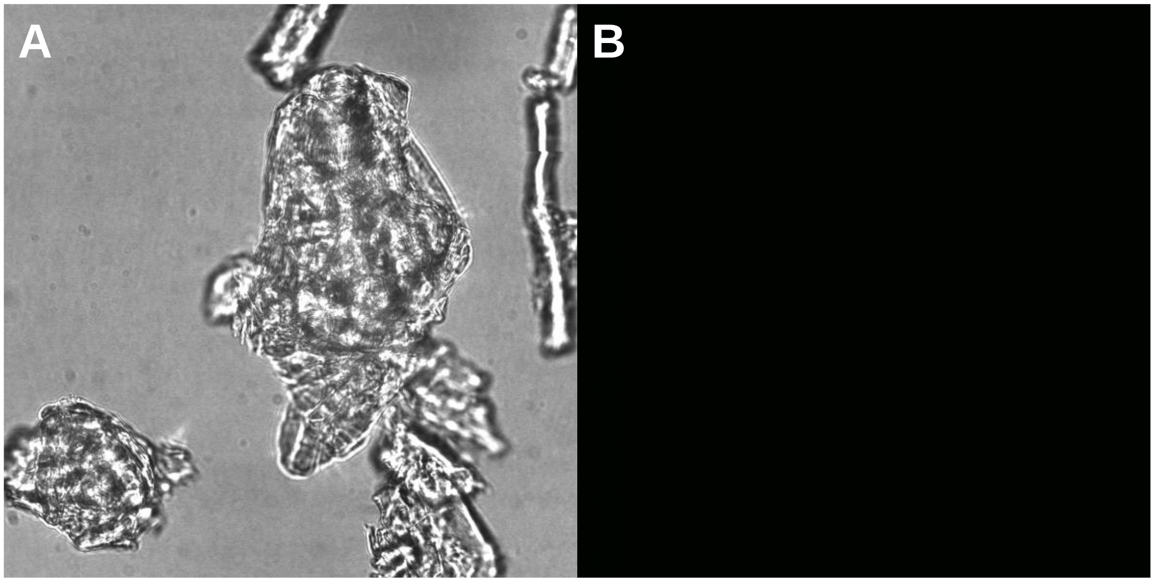

Figure S2: Brightfield (A) and confocal (B) images of untreated SA-Avicel labelled using rhodamine chloride. SA-Avicel and 1 mM of gallic acid was incubated at 50 °C for 24 h with 1 %. Insoluble products were separated, labelled with fluorescent dye, and visualized using a confocal microscope.

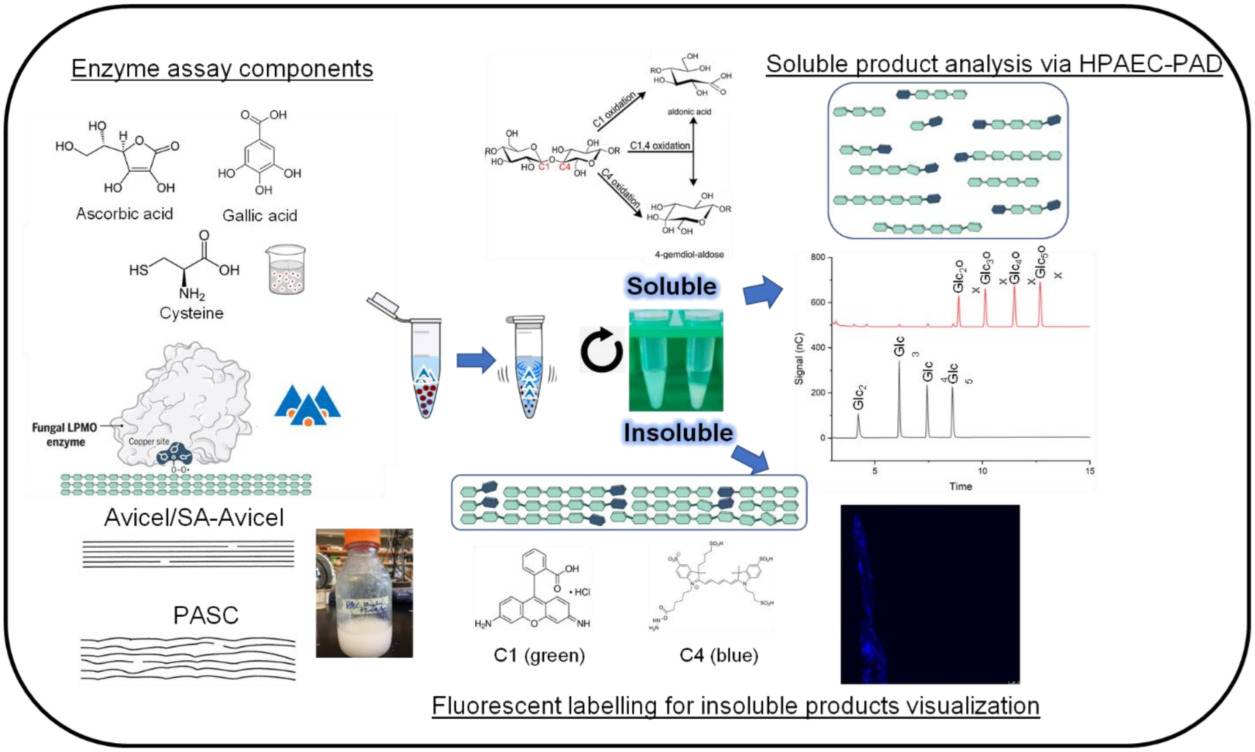

Figure S3: Process schematic for LPMO treatment of cellulose and subsequent soluble and insoluble products analysis
